# Health program for prEvention of cardiovascuLar disEases based on a risk screeNing strategy with Ankle-brachial index: HELENA study protocol

**DOI:** 10.3389/fpubh.2025.1484163

**Published:** 2025-05-30

**Authors:** Gina Domínguez-Armengol, Francesc Ribas-Aulinas, Elisabet Balló, Maite Alzamora-Sas, Mar Serrat-Costa, Anna Ruiz-Comellas, Maria Jose Forcadell-Peris, Pere Toran, Ruth Martí-Lluch, Anna Ponjoan, Jordi Blanch, Lia Alves-Cabratosa, Lluís Zacarías-Pons, Eric Tornabell-Noguera, Álvaro Sánchez-Pérez, Anna Berenguera-Ossó, Rafel Ramos, The HELENA Study Group

**Affiliations:** ^1^Institut Universitari d'Investigació en Atenció Primària Jordi Gol (IDIAP Jordi Gol), Girona, Catalonia, Spain; ^2^Network for Research on Chronicity, Primary Care, and Prevention and Health Promotion (RICAPPS), Girona, Spain; ^3^Unitat de Qualitat i Seguretat del Pacient, Atenció Primària, Institut Català de la Salut, Girona, Catalonia, Spain; ^4^Institut Català de la Salut, Generalitat de Catalunya, Girona, Catalonia, Spain; ^5^Health Promotion in Rural Areas Research Group (PROSAARU), Gerència d'Atenció Primària i a la Comunitat Catalunya Central, Institut Català de la Salut, Manresa, Spain; ^6^Centre d’Atenció Primària (CAP) Sant Joan de Vilatorrada, Institut Català de la Salut, Girona, Catalonia, Spain; ^7^Centre d’Atenció Primària (CAP) Amposta, Institut Català de la Salut, Girona, Spain; ^8^Institut d’Investigació Biomèdica de Girona (IdIBGi), Girona, Spain; ^9^Primary Care Research Unit, Deputy Directorate of Healthcare Assistance- BioCruces Bizkaia Health Research Institute, Basque Healthcare Service, Barakaldo, Spain; ^10^Department of Medical Sciences, Universitat de Girona, Girona, Catalonia, Spain

**Keywords:** peripheral artery disease (PAD), ankle-brachial index (ABI), cardiovascular risk assessment, secondary prevention, screening program

## Abstract

**Introduction:**

The use of risk functions to individualize preventive interventions is a key strategy in the primary prevention of coronary heart diseases (CHD). Unfortunately, most risk functions still fail to identify many individuals who will experience a cardiovascular (CV) event. Detecting individuals with asymptomatic peripheral artery disease (PAD) with a new risk function could improve CV risk classification. The aim is to evaluate the effectiveness of integrating an ankle-brachial index (ABI) program into the current CHD risk detection strategy to identify populations at high risk of asymptomatic PAD, reducing the incidence of CHD and mortality in those aged 50 to 74 years.

**Methods:**

This study is a pragmatic randomized cluster trial. A total of 274 primary care centers will be randomized into two groups that will either maintain the current CHD risk detection strategy or add a screening program to detect asymptomatic PAD using ABI. In routine clinical practice, 10-year CHD and PAD risk are assessed using the Framingham-adapted (REGICOR) function and the REASON function, respectively. The study population will consist of patients aged 50 to 74 years with a CHD risk ≥ 7% and PAD risk ≥ 7%, making them candidates for an ABI measurement. Cases with an ABI result ≤ 0.9 will have their CHD reclassified as high or very high by doubling the initial REGICOR score and receive the recommendations of the lipid and cardiovascular risk guideline. The primary outcomes will be hard CHD, major adverse cardiovascular events (MACE), all-cause mortality, and improvement in CVD risk factors. Secondary outcomes include CHD (a composite of angina and hard CHD), cerebrovascular disease, and adverse effects from lipid-lowering medication. Survival analysis will estimate the effectiveness of adding the ABI screening strategy, with Cox models (intention-to-treat) and marginal structural models controlling for confounding variables.

**Results and discussion:**

Direct health improvements in the intervened population are expected, including a reduction in CHD incidence and its risk factors. This project is particularly valuable, as delays in screenings and control of CV risk factors have accumulated after the COVID-19 pandemic. Therefore, this work is expected to help recover and enhance cardiovascular risk prevention efforts.

**Clinical trial registration:**

ClinicalTrials.gov, NCT05884840.

## 1 Introduction

Mortality due to cardiovascular diseases (CVD) in Spain accounted for 26.4% of all deaths in 2021; 28.9% of those occurred in women and 24.2% in men, and 24.2% were related to ischemic heart disease (1). Moreover, CVD also poses a huge economic burden: in 2021, the total cost of CVD was estimated to be €282 billion, 11% of EU-health expenditure (2).

The use of risk functions to individualize preventive interventions is a key strategy in primary prevention for CVD (3). The current coronary heart disease (CHD) screening risk function in some regions of Spain is the Framingham-adapted REGICOR (*Registre Gironí del Cor*) function (4). This function is integrated in the primary care electronic health records (EHR) system and predicts the probability of developing a coronary event within 10 years (5). Those with more than 10% of such probability are at high-risk, and thus, candidates for treatment with lipid-lowering medication and recommendations on healthy lifestyles. However, studies have also described that many events occur in people classified as having moderate risk (5–9.9%), who are not yet candidates for treatment (5, 6).

The current primary prevention strategy proposes additional (bio)markers to improve CHD risk classification (7, 8). A good CHD (bio)marker meets some basic criteria: (a) it is easily measurable and has a relatively high prevalence, (b) it is an independent predictor of CHD, and (c) when evaluated, it should classify a substantial proportion of the population who will suffer cardiovascular (CV) events as being at high risk (9). Among these (bio)markers, those that identify the presence of asymptomatic atherosclerosis are the most reliable to detect patients in the highest risk group (3, 9, 10).

The ankle-brachial index (ABI) is one of the best candidates that meet the above-mentioned three criteria (7, 8). The ABI can be easily measured with a non-invasive and inexpensive technique. Values lower than 0.9 are used to diagnose lower extremity artery disease [henceforth referred to as peripheral artery disease (PAD), which is associated with higher mortality risk and CV events (11)]. The ABI values provide independent risk information additional to coronary risk functions (7). A meta-analysis showed that having an ABI below 0.9 doubled the 10-year total mortality, CV mortality, and the risk of a major coronary event (MACE) (7). As noted by Poredoš et al., a low ABI is a useful screening tool that can be helpful in re-classifying patients’ CV risk into higher categories (12). Furthermore, a more recent local study performed in the province of Barcelona, the ARTPER study, also reported that the presence of PAD doubles, at a minimum, the risk of MACE as well as the risk of coronary disease and vascular mortality (9). Importantly, another local study in Girona (a region of Catalunya) reported that 86% of people with an ABI ≤ 0.9 did not exhibit any symptom (13).

Even though the ABI test is a simple technique and is performed regularly in the primary care settings, it is also time-consuming and requires devices and trained personnel to ensure accurate measurements (13). The inter-society consensus (ISC) practice guidelines for the management of people with PAD recommend ABI screening in patients aged 50–70 who have diabetes or are smokers, and in patients older than 70 years (14). However, these recommendations have a limited level of evidence, and the precision of PAD detection in the asymptomatic population has been hardly studied.

Therefore, we designed a new risk function, derivation of the REgicor and Artper Score fOr aNkle brachial index screening (REASON) (15), to select the best candidates for an ABI measurement. This model improved the prediction of ABI ≤ 0.9 in Spanish patients aged 50–74 who were apparently free of CHD (16), and showed similar sensitivity and improved specificity compared to ISC. Furthermore, the predictive ability of REASON can be modulated by changing the cut-off risk point. This feature can be useful to manage the available resources for CV prevention in each region of the world (15). A cut-off point of 7% has a sensitivity of 68%, a specificity of 72%, and selects 35% of the population as candidates for ABI screening (15). This improvement in predictive capacity translates into a considerable reduction in the number of screened people needed to detect a case with ABI ≤ 0.9. Therefore, by reducing the number of false positives and maintaining the sensitivity, the use of REASON should limit the workload and possible adverse events associated with CHD diagnostic procedures and medical treatments (15).

The adoption of a strategy that combines the current CHD risk estimation with REGICOR and the probability of having an ABI ≤ 0.9 assessed with REASON would detect patients that require an ABI test and improve the CHD risk classification (7, 8, 17). The diagnosis of asymptomatic PAD at an early stage can be a critical opportunity to influence the disease progression, prevent its complications, and control its risk factors. Additionally, early diagnosis can also be a motivation to help patients accept the recommendations and improve lifestyle habits, such as smoking cessation, diet, and increased physical activity.

The study that measured the predictive capacity of REASON for ABI ≤ 0.9 was observational, and thus, further research is needed to validate its results (15). Consequently, we have designed this clinical trial to appraise the effectiveness of REASON:

The aims of this study are: (1) to evaluate the effectiveness of integrating an ABI screening program into the current CHD risk assessment strategy for reducing the incidence of CHD and all-cause mortality among individuals aged 50 to 74, and (2) to evaluate the effectiveness of integrating an ABI screening program into the current CHD risk assessment strategy for mitigating CV risk factors among individuals aged 50 to 74.

## 2 Methods

### 2.1 Study design

This study will be conducted as a pragmatic cluster randomized trial in the primary care centers (PCC) of Catalonia. Patient monitoring will take place during a period of 2 years (2023–2025).

### 2.2 Participants

We will include people aged 50 to 74 years who had a REGICOR score ≥ 7% and a REASON score ≥ 7%, during a routine primary care visit. The exclusion criteria are patients with a previous history of symptomatic PAD, coronary disease, stroke, cardiac revascularization, or cLDL≥ 190 mg/dL or equivalents.

### 2.3 Sample size

The incidence of CV events (coronary or cerebrovascular disease, or symptomatic PAD) is expected to be 7% in the selected population during a period of 3 years. Accepting a 0.05 alpha risk and a 0.2 beta risk in a bilateral contrast, 20,171 people are needed in each arm, intervention and control groups, to detect a relative risk of 0.9.

Around 500.000 people aged 50–74 years have their CHD risk calculated in Catalonia per year. Out of those, we estimate that 36.000 will present both a REGICOR and REASON score ≥ 7%. Thus, in one and a half years we expect that there will be 54.000 people, 27.000 for each arm.

### 2.4 Intervention

This intervention aims to integrate an ABI screening program into the current CHD risk assessment in the primary care setting. We will use the prediction capacity of the REASON risk model to select the best candidates for ABI measurement in individuals aged 50–74 without previous CVD.

**Figure 1:**
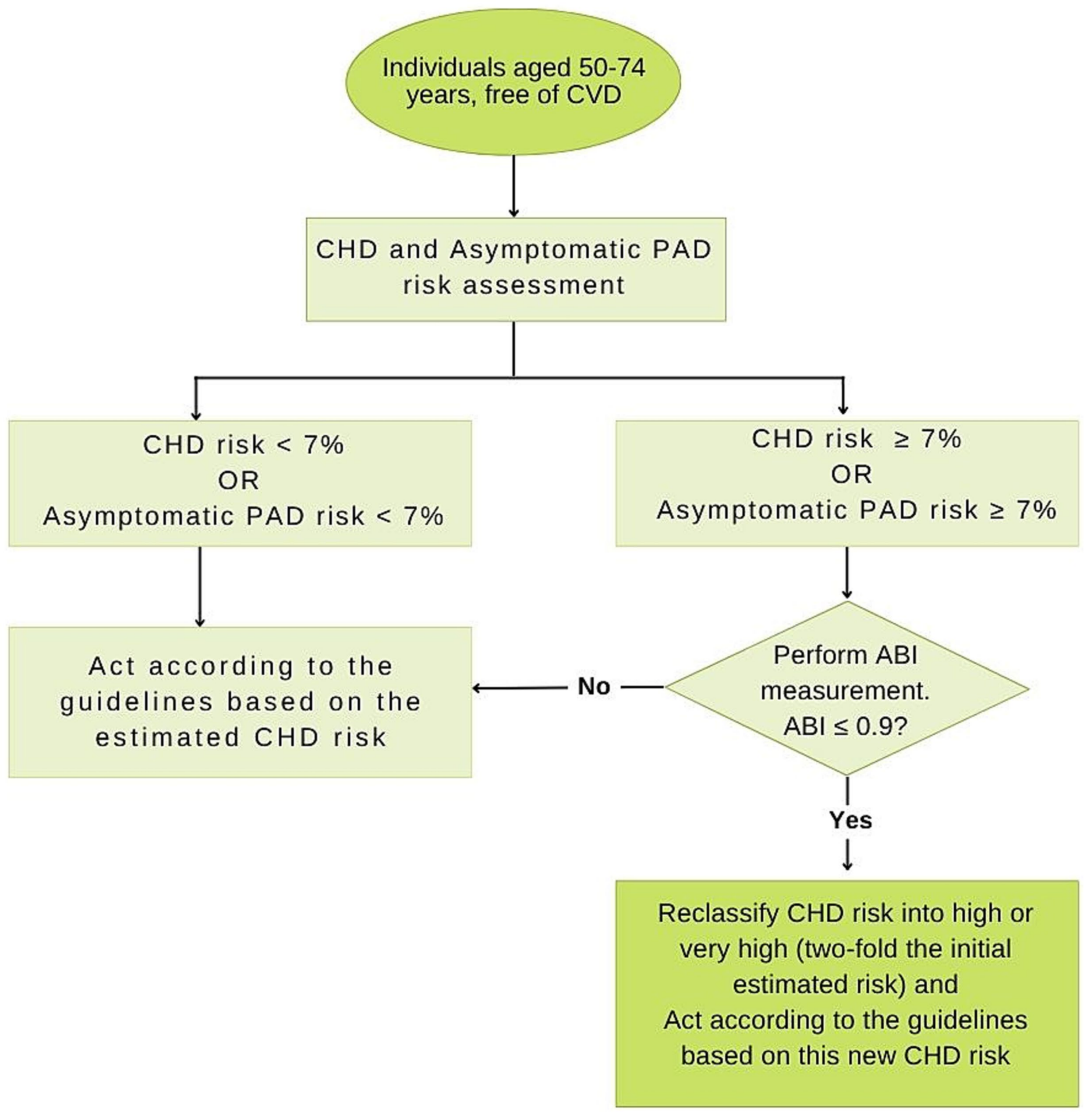
Diagram of the HELENA study protocol. CHD, Coronary heart disease; ABI, Ankle-brachial Index; PAD, Peripheral artery disease.

The whole intervention process is illustrated in Figure 1. During a routine primary care visit, individuals who meet the inclusion criteria will undergo the CHD risk assessment with REGICOR. If their REGICOR score is ≥ 7%, we will calculate their probability of having a low ABI, ≤ 0.9, using the REASON risk function. Individuals with a predicted probability of low ABI ≥ 7% will be the candidates for an ABI measurement performed by a trained health professional.

The CHD risk of individuals with ABI results ≤ 0.9 will be reclassified. According to a previous meta-analysis, individuals with an ABI ≤ 0.9 have at least twice the 10-year total mortality, CV mortality and major coronary event rates compared to individuals with normal ABI values (7). Therefore, in individuals with an ABI ≤ 0.9, the initial REGICOR risk score will be doubled, and the patient reclassified into a high or very high CHD risk group.

Subsequently, physicians will provide recommendations based on the Catalan Health Institute’s Guidelines about Lipids and Cardiovascular Risk (18). For asymptomatic patients with ABI ≤ 0.9, the recommendations are as follows: (1) healthy lifestyle modifications: encourage healthy lifestyles such as regular physical activity (at least 30 min per day of moderate physical activity), adhesion to Mediterranean diet reduction of saturated or trans fats, daily consumption of fruits and vegetables, and consumption of fatty fish 2 days per week, weight management, reduction of alcohol consumption, and smoking cessation; (2) medication: use of moderate-intensity statin treatment based on individual CHD risk score. For high CHD risk score use simvastatin 20 mg/dL, atorvastatin 10 mg/dL, or pravastatin 40 mg/dL. For very-high CHD risk score, use simvastatin 40 mg/dL, or atorvastatin 20 mg/dL; (3) control of CVD risk factors: hypertension, diabetes, and hyperlipidemia. Patients with ABI > 0.9 should follow the recommendations of the guidelines based on the estimated CHD risk (Figure 1).

#### 2.4.1 Pilot study

To assess key factors that could challenge the intervention implementation or would ensure its success, we conducted a pilot study over 2 weeks in March 2023 in 17 primary care centers. This study was supported by a group specialized in implementation research. Data on factors that ensured a successful implementation were collected via questionnaires and focus groups from health professionals. Participants in this pilot highlighted the importance of being familiar with the Catalan Health Institute’s Guidelines on Lipids and Cardiovascular Risk, and acquiring the necessary skills for the new screening program. Health professionals also emphasized the importance of effectively conveying the benefits of the screening program. Moreover, effective planning and organization within each center were identified as pivotal factors for the implementation of the intervention.

This information was used to develop a strategy for the adoption of the new CHD screening program. The strategy was based on the following main elements: (1) commitment to a common goal: a series of workshops and training sessions were organized to raise awareness of the new procedure; a representative of the health professionals was designated in each center to emphasize the life-saving potential of the new screening strategy; (2) collaborative organizational action: each center was encouraged to define a workflow considering the workload of the professionals and their available resources; (3) need for tension to prompt change: regular feedback loops were established to discuss project challenges and early results; performance monitoring variables were also integrated into the EHR and reviewed during the feedback sessions to maintain collective commitment; (4) sustainability of the intervention over time: to ensure sustainability, periodic sessions and updates were planned with the representatives of each center.

### 2.5 Data collection and management

This is a pragmatic clinical trial. Basic and monitoring data will be generated from the regular clinical practice; afterwards, the generated data will be extracted from the EHR. The following data will be collected:

#### 2.5.1 Cardiovascular risk variables

We will use the REGICOR’s and REASON’s function scores; and the variables used for its calculation: age, sex, systolic and diastolic blood pressure, diabetes, tobacco consumption, total and HDL cholesterol; and ABI’s result if it is measured.

#### 2.5.2 Monitoring and result variables

Monitoring of the patients’ data will occur every 3 months during the first semester of the study to assess the implementation. After that, it will be conducted every 6 months up to 3 years from the patients’ study enrolment.

The study primary outcomes will be hard CHD (myocardial infarction, cardiac revascularization, or coronary death); major adverse cardiovascular events (MACE), a composite of hard CHD and stroke (fatal and nonfatal ischemic stroke); all-cause mortality; and variables related to the assessment of CVD risk factors improvement: tobacco consumption, lipid profile, systolic and diastolic pressure, weight, height, body-mass index (BMI), glycemia, glycated hemoglobin (in patients with DM), creatinine, proteinuria, albumin-to-creatinine ratio, and glomerular filtration rate.

The secondary outcomes will be CHD (a composite of angina and hard CHD), cerebrovascular disease (a composite of stroke [fatal and nonfatal ischemic stroke] and transient ischemic attack); CVD (a composite of MACE, angina, and transient ischemic attack); lipid lowering medication adverse effects: short-term effects (muscular and hepatic alterations) and long-term effects (diabetes and cancer).

The considered potential confounding variables included sociodemographic information: age and sex; PHC related variables: attendance rate, billing rate, and length of time in the EHR; alcohol consumption; comorbidities: diabetes, atrial fibrillation, heart failure, dementia, endocrine and metabolic conditions, inflammatory diseases, asthma, chronic obstructive pulmonary disease, depression, CVD incidence rate; other comorbidities identified in the bivariant analysis; used medication: corticosteroids, antidepressants, hormonal substitutive therapy, oral contraceptives, antipsychotics, anti-inflammatories, Platelet aggregation inhibitors (other than heparin), antihypertensives, anti-diabetic drugs, and lipid lowering medication; laboratory related variables: alanine transaminase (ALT) and aspartate aminotransferase (AST). We will also record health-care quality standards of health care providers and a deprivation index (MEDEA index) (16).

### 2.6 Statistical analysis

Quantitative continuous variables will be described with the mean and standard deviation or with the median and the interquartile range; categorical variables will be described with frequencies (%). Continuous variables will be compared using the Student’s t-test or Analysis of variance (ANOVA); Mann–Whitney U or Kruskal-Wallis will be applied when pertinent. Categorical variables will be compared using the Chi-squared or Fisher exact test as needed.

Survival analysis tests will be used to estimate the effectiveness of introducing a screening program using ABI. We will build classical Cox models and marginal structural models to account for confounding and intermediate variables. The reduction of the absolute risk and the number needed to treat (NNT) to prevent one additional event will also be calculated.

### 2.7 Ethical considerations

This study has received the ethical approval from the SIDIAP Scientific Committee and the Research Ethics Committee from the Institut d’Investigació en Atenció Primària Jordi Gol i Gurina (reference number 22/088-P). The researchers are committed to respect the principles of human experimentation and good medical research from the Declaration of Helsinki and the 17.2.d Spanish “Organic Law 3/2018, 5th of December, personal data protection, and digital rights guarantee.” The data for analysis will be sourced from pseudonymized electronic medical records and will only be available to the research team, which will sign a confidentiality agreement before obtaining them.

## 3 Results and discussion

Cardiovascular diseases are the leading cause of death and disease burden worldwide. They have been associated with 6.2 million deaths in people aged 30 to 70 years in 2019 (19). The strategies to reduce this burden include the promotion of healthy lifestyles to improve behavioral risk factors and opportunistic screening in primary care facilities (20).

The introduction of this new PAD screening program using the ABI ≤ 0.9 predictive function will directly benefit the target population. The diagnosis of asymptomatic PAD at an early stage can be crucial for patient motivation and encourage acceptance of medical recommendations. This creates an opportunity to prevent disease progression, control risk factors, and improve lifestyles through smoking cessation, exercise, and diet.

The US Preventive Services Task Force stated that the available evidence is insufficient to establish population-based screening programs for PAD (20). However, this conclusion has been controversial because it did not consider the potential for preventing not only the progression of PAD itself, but also the development of severe CV events (17).

Moreover, a previous study, the Viborg Vascular Screening Trial (VIVA Study), evaluated the effectiveness of a combined screening strategy that included detection of PAD with ABI, detection of abdominal aneurysm, and blood pressure screening in men aged 65–74 years. After a 4.4-year follow-up, they observed a relative reduction in mortality of 7% in the intervention group, which translated into an absolute reduction of 0.6% (21). However, this study combines three interventions, which did not allow the evaluation of the screening with ABI alone. Besides, the study population included only men aged 65–74 without any criteria regarding CV risk. Another two ongoing trials also include ABI as part of their screening, but not in isolation (21, 22). The US Preventive Services Task Force does emphasize the need for conducting large trials to assess the effectiveness PAD screening using ABI measurement (20). These studies, in addition to isolating the effect of individual tests, should target individuals with an elevated risk of PAD who would not yet be receiving interventions to reduce their CHD risk (20). This is the population most likely to benefit from a screening intervention (20).

In our area, published evidence supports the effectiveness of preventive measures in individuals with asymptomatic PAD and no clinical CVD (23). In a previous study, we found that statin therapy was associated with a reduction in CV events and mortality in such a population, regardless of their CHD risk (23). The absolute reduction was comparable to that achieved in secondary prevention (23).

We expect that the impact of this intervention will not be limited to only CVD, but also other conditions related to lifestyle behaviors, such as reduction of smoking habits, increase in physical activity, and adhesion to Mediterranean diet. Importantly, the introduction of an ABI screening strategy into the current CHD risk assessment, is a groundbreaking approach to improve the CHD risk assessment. This innovative strategy is easy to perform and the technique included is non-invasive. Moreover, it aims to detect asymptomatic patients, thus becoming a useful risk modifier that will seek earlier interventions and overall improvement in health outcomes. This proposal offers a great opportunity, even more so since the COVID-19 pandemic has led to a reduction of CVD diagnoses at the primary care level. We expect that this strategy will contribute to restore and enhance CVD screening and prevention in the primary care settings (24).

### 3.1 Limitations

A major challenge of this study could have been the implementation of the intervention, but this has been addressed beforehand. We conducted a detailed implementation pilot study with the support of a group specialized in this field. Another limitation could be the quality of the data. However, we expect to minimize this challenge using the EHR from the primary care information systems. These records have been used extensively in research and validated (25), including the validation of medication exposure, which will be confirmed through the billing records from the community pharmacies. We also acknowledge that the success of the results of this project will rely on participants’ adherence to the treatment and recommendations after being classified as high risk. Measures to address this limitation have been carefully implemented through training, the provision of educational materials, and the designation of appointed persons in each primary care center to raise awareness on PAD and its importance. Finally, extrapolation of results to a broader population or other demographic groups should be cautious. The aim of this study is to include the ABI measurement to refine the current CHD screening program of the population classified as medium CHD risk and aged 50–70 years old.

## Group members of The HELENA study group

Natalia Maella Rius, Judith Isach Morato, Janina Valls Pascual, Pilar del Castillo Sanchez, Carolina Rodríguez Sardañes, Patricia Fernández Díaz-Flores, Lara Martínez Real, Ester Serrano Serrano, Sara Casado Cortés, Ona Alomar Morillo, Xenia Garcia Nuñez, Mª Pilar Gea Martinez, Guadalupe Layos García, Avelina Hidalgo Arenas, Laia Mas Pons, Laia Briansó Castilla, Silvia Torres Santaló, Ada Pallares Gorriz, Hajar El Jaouhari Khlifi, Fina Aranda Valero, Judith Company Fontané, Carmen López Serral, Rosario Martínez Garrido, Marta Sánchez Corbalan, Aida Fortuny Borsot, Mercè Iruela Sanchez, Cristina Teixido Vargas, Marta Pérez García, Marta Montoza Serrano, Carme Blanco Aguilar, Ana Mª Cosmen De Paz, Cristina Brunell Sauque, Marta Requena Saez, Isabel Carretero Huertas, Marisol Rubio Rodriguez, Brigitte Banessa Galagarza Teran, Esteve Sanchez Morillo, M Carmen Danta Gomez, Maria Julia Guiteras, Montserrat González Lluis, Jordi Salas Palazon, Núria Salarich Sola, Leonor Jimenez Massana, M. Pilar Martin Zafon, Anna Patricia Perez Salvador, Josep Canyelles Ballús, Susana Carrasco Turigas, Monica Espelt Llorens, Miriam Lara Rodriguez, Marta Urquizu Rovira, Cristina Mansilla Garcia, Lidia Borras Español, Marta Blanco Mena, Montserrat Pons Segura, Eva Madurell Vera, Irene Muñoz Garcia, Laura Coletes Llorens, Lourdes Gil Marti, Eva Jaimez Sanjuan, Maria Josep Lopez Gomez, Marina Redondo Vidal, Ana Maria Escayola Maranges, Anna Corrales Mas, Monica Garcia Mañero, Raquel Planas Simon, Saray Ruiz Ortiz, Meritxell Virgós Soler, Yana Stoyanova Gunderova, Ildefonso Casais Padilla, Esther Soria Martin, Paz Martí Romero, Teresa Mur Marti, Mª Rosa Jurado, Sandra Rojas Torres, Carmen Frutos Fuentes, Luisa Avilés Gonzalez, Montse Muñiz, Ana Arroyo fernandez, Anna Monforte Batiste, Maria Nogué Font, Marta Roura Fausellas, Eva Maria Arco Avila, Àngels Rodríguez Calonge, Montserrat Gosalbez Götzens, Iris Forcada Parrilla, Marta De la Barrera Pujol, Minea Torres Gestoso, M. Jose Palacios Gordillo, Montserrat Jover Mallol, Joan Roca Seoane, Mireia Barat Gonzàlez, Clara Sendra Ortolà, Marc Ribas Aulinas, Laura Codina Pena, Núria Lozano Martí, Elisenda Santiveri Torras, Mireia Bravo Sanchez, Nelson Urra Andarsio, Marta Perez Garcia, Sílvia Joher Sabat, Mónica Rios Cuevas, Miquel Sitjar Suñer, Erica Homs Romero, Enric Estévez Rovira, Leonardo Heredia Rodríguez, Pyrene Martínez, Susanna Trèmols, Imma Morató Fornaguera, Daniel Sánchez, Mireia Prat, Gemma Rico Rota, Anabel Lancho Medina, Irma Fornell Boixader, Maria Josep Rodriguez Moran, Marc Cases Ratés, Yolanda Maria González Fenández, Gloria Fitó Guillemas, Nuria Cerarols, Maria del Carmen Reyes, M Olga Llamazares Robles, Bàrbara Azuaga Martín, Anna Cervera Steegmann, Loubna Alami Bazza, Emilia Mateo Marin, Sandra Simó Llavero, Núria Gordo Serra, Anna Coma Roura, M. Carme Parareda Plana, Magda Tarres Nogués, J.M. Laliena Carol, Eva Codinach Danes, Judit Cañada Puigdomenech, Raquel Vioque Puebla, Gloria Arnau Sole, Jordi Casanovas, Josep Maria Vilaseca Llobet, Josep Aguilar Urpí, Roxana Catalin, Laia Gené Huguet, Joaquín Breva Aymerich, Mònica Torres Jiménez, Magdalena Garcia Olive, Albert del Pozo Niubó, Julio Andres Viñales Berna, Sandra Navarrón Rojo, Laura Ruiz de Porras Rosello, Judith Mateo Rovira, Joan Lluís Frigola Marcet, Pablo Hsu Chung, Maria Moreno Gómez, Ester Granado Font, Elisabet Masip Bartolomé, Domingo Ribas Seguí, Antoni Santigosa Ayala, Begoña Segura Cañada, María Juana Serra Virgili, Assumpta Gonzalez Garces, Miriam Boira Costa, Ramon Descarrega, Jose Ribó, Glòria Carol, David Garcia, Rosa Rovira Anglès, Sònia Loran Varcárcel, Anna Bibiloni Solé, Aida Pentinat Pelegrin.
